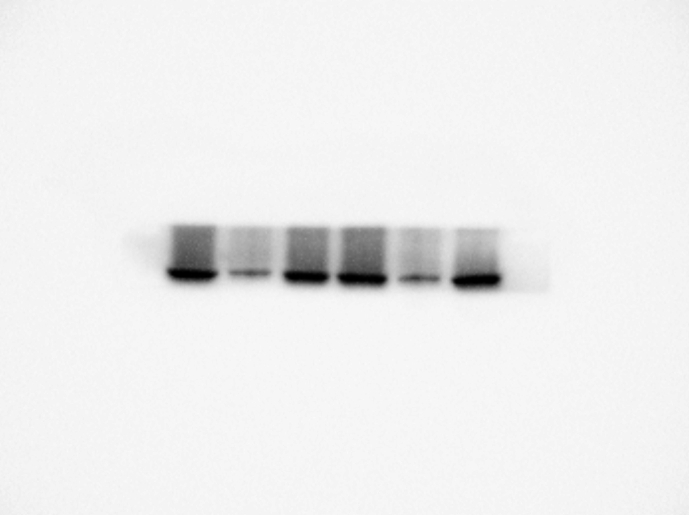# C1GALT1 induces the carcinogenesis of thyroid cancer through regulation by miR-141-3p and GLUT1

**DOI:** 10.1016/j.heliyon.2024.e31778

**Published:** 2024-05-24

**Authors:** Li Huang, Zhen Li, Ziguang Xu, Ruili Yu, Chao Ding, Tingyi Sun, Lingfei Kong, Zhengchao Xia

**Affiliations:** a Department of Pathology, Henan Provincial People's Hospital, Zhengzhou University People's Hospital, Henan, China; b Department of Thyroid, Henan Provincial People's Hospital, Zhengzhou University People's Hospital, Henan, China; c Department of Pharmacy, Henan Provincial People's Hospital, Zhengzhou University People's Hospital, Henan, China

**Keywords:** Thyroid cancer, C1GALT1, miR-141-3p, GLUT1, Carcinogenesis, Metastasis

## Abstract

Core 1 β 1,3-galactosyltransferase 1 (C1GALT1) acts as an important glycosyltransferase in the occurrence and development of tumor glycosylation. However, the regulatory mechanisms of C1GALT1 in thyroid cancer (TC) is still unclear. In this study, we discovered that the expression level of C1GALT1 was significantly increased in thyroid adenocarcinoma tissues and cell lines (*p* < 0.01). Meanwhile, gene silencing of C1GALT1 inhibited the proliferation (CCK-8 assay), migration (wound healing), and invasion (Transwell) of TC cells (*p* < 0.05). Further investigation indicated that miR-141-3p had a negative correlation with C1GALT1 and suppressed cancer carcinogenesis in TC cells. Moreover, we first found that glucose transporter 1 (GLUT1) was a downstream element of C1GALT1 and was positively correlated with C1GALT1 levels in TC. The GLUT1 could reverse the inhibitory effects of siRNA C1GALT1 on cell development (*p* < 0.05). These data suggest that the miR-141-3p/C1GALT1/GLUT1 axis plays an essential role during TC progression and may be a probable biomarker or therapeutic target for thyroid cancer patients.

## 1 Introduction

The incidence of thyroid cancer (TC) has been increasing significantly worldwide and has become one of the most common endocrine system tumors. Approximately 77 % of thyroid cancer patients were women, with an incidence more than double that in men [1^]^. According to global cancer statistics in 2020, TC has become one of the top 10 malignant tumors in the world, especially in the top 3 female malignant tumors [1,2]. In recent years, the main diagnostic methods of TC have included imaging and pathological examination [3]. Although therapeutic strategies for TC have been perfected, some patients have a high risk of recurrence or metastasis within 5 years after surgery [4,5]. Therefore, it is essential to elucidation the pathogenesis of TC, which conducive to provide new therapeutic targets and improve therapeutic effect.

Glycosylation is the most ubiquitous posttranslational modification in nature and plays an important role in protein structure and function. Glycosylation reactions, mostly catalyzed by glycosyltransferases, are almost universal in tumor evolution [6,7]. To date, approximately 300 glycosyltransferases have been identified according to the carbohydrate active enzyme database (www.cazy.org). Glycosyltransferase is closely related to the pathological process of tumor. Glycosyltransferases combine low molecular sugar, such as galactose or mannose, with different amino acids to form glycans, which affect protein modification functions [8]. O-linked and N-linked glycosylation are the major types of glycosylation [9,10]. Recent studies have revealed that the Thomsen-nouvelle (Tn) antigen is catalyzed by α-N-acetylgalactosaminyltransferase, which binds GalNAc to specific serine or threonine residues. Then, the extension of Tn antigen further forms O-glycan, core 1-derived structures (T antigen) [11,12]. Core 1 β 1,3-galactosyltransferase 1 (C1GALT1, T-synthase) is the key enzyme in the process of core 1-derived O-glycans. Studies shown that C1GALT1 is abnormally expressed in a variety of malignant tumors, such as colorectal cancer [11], gastric cancer [13], pancreatic adenocarcinoma [14] and lung cancer [15]. However, the role of C1GALT1 in thyroid cancer remains unclear, and the pathogenesis has not been elucidated.

In this study, we comprehensively assessed the regulatory mode of C1GALT1 in TC using molecular biology technology and bioinformatics tools. We found that C1GALT1 was overexpressed in TC tissues and cells. Through mechanistic studies, we further confirmed that C1GALT1 could promote TC progression. C1GALT1 was also negatively regulated by miR-141-3p and positively mediated glucose transporter 1 (GLUT1) expression in TC. These results may provide a novel prognostic biomarkers and potential therapeutic targets for TC.

## 2 Materials and methods

### 2.1 Cell culture

The human thyroid cancer cell lines BCPAP and TPC-1 were purchased from the National Collection of Authenticated Cell Cultures (Shanghai, China). All cells were cultured in Roswell Park Memorial Institute (RPMI) 1640 (12633012, Gibco, USA) supplemented with 10 % fetal bovine serum (FBS, 10100, Gibco, USA) and 1 % penicillin/streptomycin (15140122, Gibco, USA) at 37 °C in a 5 % CO_2_ humidified incubator.

### 2.2 Ethical approval

This study was approved by the Ethics Committee of Henan Provincial People's Hospital, with the ethics approval number: 2021-84. Written informed consent was obtained from all the participants. Clinical specimens were collected from Henan Provincial People's Hospital. All patients were chemotherapy and radiation therapy naive. The samples were immediately frozen in liquid nitrogen and stored at −80 °C.

### 2.3 Cell transfection

The C1GALT1 small interfering RNA (siRNA), GLUT1 overexpression plasmid, miR-141-3p mimics and their corresponding negative control (NC) were purchased from Sangon Biotech (Shanghai, China) and RiboBio (Guangzhou, China), respectively. Cell transfection was performed using Opti-MEM lower serum medium (31985070, Gibco, USA) and Lipofectamine 2000 (11668, Invitrogen, USA). The transfection efficiency was identified by real-time quantitative polymerase chain reaction (RT-qPCR) and western blotting assays. The sequences of the siRNAs are listed in Supplementary Table S1.

### 2.4 Lectin microarray analysis

Protein glycosylation was conducted by lectin microarray analysis containing 56 lectins. The experiment was completed by BC Biotechnology (Guangdong, China). In brief, the protein of clinical specimens was extracted, labeled by an EZ-Link Sulfo–NHS–LC-Bioto kit (21335, Thermo Scientific, USA), blocked and incubated with Cy3-streptavidin (Cy3-SA, S6402, Sigma, USA). The image and data were produced using a GenePix 4200 scanner and GenePix Pro v6.0 software (Molecular Devices, USA) [16^]^.

### 2.5 Real-time PCR

Total RNA was isolated from clinical samples or cell lines using TRIzol. The detailed experiments of reverse transcription and quantitative polymerase chain reaction were performed as described in a previous study [16]. For miRNA analysis, the TaqMan miRNA Reverse Transcription Kit and miRNA Assay Kit (Applied Biosystems, USA) were used. The expression levels were calculated using the 2^−ΔΔct^ method after normalization to the expression of GAPDH or U6. The sequences of all primers used for PCR are listed in Supplementary Table S1.

### 2.6 Lectin blotting and western blotting

Total protein was extracted by RIPA, quantified by BCA Assay Kit, separated by SDS-PAGE, transferred to PVDF membranes, and incubated with different antibodies for Western blot. The blots were visualized with the PXi 9 chemiluminescent detection system and ImageJ [17^]^. A list of antibodies is provided in Supplementary Table S2.

For lectin blot analysis, the membranes were blocked with carbo-free blocking solution (Vector Labs, Burlingame, USA) and incubated with biotinylated peanut agglutinin (PNA) overnight at 4 °C. Subsequently, HRP-conjugated streptavidin was used as a secondary antibody for 1 h. The remaining processes of blots were consistent with western blotting.

### 2.7 C1GALT1 activity assay

C1GALT1 activity was measured as described in a previous study [11^]^. In brief, tissue extracts were prepared by homogenization buffer. Then, supernatant protein sample and master mix were added to an opaque black 96-well plate, incubated at 37 °C for 1 h. Finally, the fluorescence intensity was assayed by SpectraMax i3 microplate reader (excitation wavelength: 355 nm and emission wavelength: 460 nm). A reaction mix without UDP-Gal was used as control.

### 2.8 Cell proliferation assay

This assay was conducted by Cell Counting Kit-8 (CCK-8). Cells (1 × 10^4^ cells/well) were seeded into 96-well plates and added CCK-8 solution at 4, 24, 48 and 72 h after transfection. The 96-well plate was incubated at 37 °C for 2 h. The absorbance was detected by a microplate reader (Bio-Rad, USA) at 450 nm. These experiments were performed in triplicate.

### 2.9 Cell migration and invasion assays

The protocols of cell migration and invasion assays were identical to those described previously [17^]^. Briefly, 1 × 10^5^ cells were cultivated (without FBS) for 24 h in a 6-well plate for the migration assay. The wound healing of scratches was observed by microscope camera at 0 and 24 h after treatment with pipette tip. For the cell invasion assay, Matrigel diluent was coated into the upper compartment of the chamber. Then, the upper compartment was seeded with 5 × 10^4^ cells following Matrigel solidification. After incubation, the cells in the upper chamber were removed with a cotton swab, fixed with paraformaldehyde, and dyed with crystal violet. Photos were also acquired by microscope camera. All assays were independently repeated in triplicate.

### 2.10 Dual-luciferase reporter assay

The pmirGlo vectors of the C1GALT1 wild-type (WT) or mutant (MUT) 3′UTR were designed and synthesized by GenePharma (Shanghai, China). The has-miR-141-3p mimics or NC mimics were cotransfected into 293T cells using Lipofectamine 2000 with the above reporter vectors. After transfection for 48 h, the luciferase activity was measured using the Dual-Luciferase Reporter Assay System (Promega, USA).

### 2.11 Detection of targeted energy metabolites

All metabolites were detected by MetWare based on the ultra-performance liquid chromatography (UPLC)-MS/MS platform (AB Sciex QTRAP 6500, Wuhan, China). The full detailed methods are described in the Supplementary Materials. In short, the samples were freeze-thawed 3 times with methanol in liquid nitrogen or ice. Then, the supernatant was used for UPLC-MS analysis after protein precipitation. Through metabolite identification and bioinformatics processing, significantly regulated metabolites were screened by variable importance in projection (VIP), *p* value and absolute log_2_FC (fold change). Functional annotations and pathway enrichment analysis were performed using the Kyoto Encyclopedia of Genes and Genomes (KEGG) and HMDB databases.

### 2.12 Statistical analysis

Data were analyzed using GraphPad Prism 9 and presented as the mean ± standard deviation (SD) (GraphPad Software, USA). Student's *t*-test, one-way ANOVA and Kaplan-Meier (KM) survival analysis were used for comparisons between two groups or multiple groups. *P* < 0.05 indicated statistical significance.

## 3 Results

### 3.1 C1GALT1 is overexpressed in thyroid cancer tissues

The 56 lectins with different glycan-binding preferences were obtained by lectin microarray analysis of clinical specimens to address aberrant glycosylation in thyroid cancer (Supplementary Fig. S1). The heatmap shows different lectins between tumor group and normal group in Fig. 1A. Meanwhile, we found that three lectins, ulex europaeus agglutinin I (UEA-I), vicia villosa lectin (VVL), and PNA, exhibited stronger binding affinities to tumor tissues with filtering of fold change and *p* value (Fig. 1B–Table S3).

**Fig. 1:**
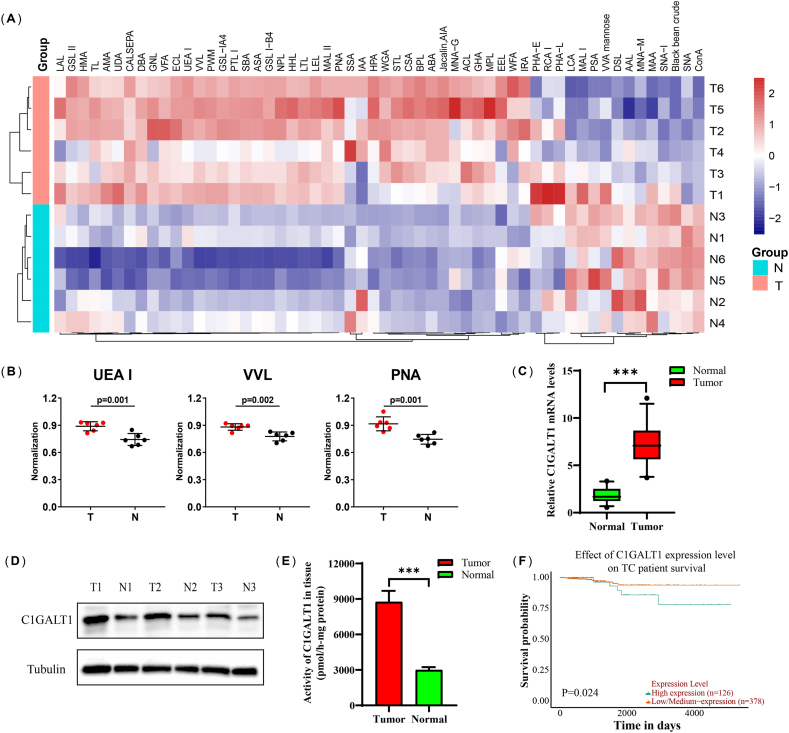
C1GALT1 is overexpressed in thyroid cancer tissues. **(A)** Heatmap of 56 lectins in the microarray analysis (n = 6). **(B)** The lectin expression levels of UEA-I, VVL and PNA. **(C, D)** C1GALT1 expression at the mRNA level (n = 30) and protein level (n = 6). **(E)** The enzyme activity of C1GALT1 in clinical specimens (n = 30). **(F)** The survival probability of different C1GALT1 expression levels in TC patients. The data are presented as the mean ± SD. ***p < 0.001.

It has been reported that PNA can bind to the T antigen, which is a special intermediate product of O-linked glycosylation, while C1GALT1 plays a crucial role in subsequent O-linked glycosylation [17]. To further identify the expression levels of C1GALT1, we detected that the mRNA and protein levels of C1GALT1 were higher in TC tissues (Fig. 1C and D, S7). The enzyme activity assay also indicated higher expression levels in the tumor group (Fig. 1E). As shown in Fig. 1F, KM survival analysis illustrated that the high expression level of C1GALT1 in TC patients presented shorter overall survival (*p* < 0.05).

### 3.2 C1GALT1 expression levels in TC cells

The mRNA expression profile of C1GALT1 showed high expression in 16 TC cell lines from the Cancer Cell Line Encyclopedia (CCLE) database (Fig. 2A). To elucidate the functional role of C1GALT1, BCPAP and TPC-1 cell lines were pretreated with C1GALT1 siRNAs for 72 h. These results confirmed that C1GALT1 decreased with siRNA1 (S1) and siRNA (S2) pretreatment at the mRNA and protein levels (Fig. 2B and C, S8). Meanwhile, the result of the lectin blotting assay verified the microarray analysis. Lower PNA binding was observed in the C1GALT1 siRNA group, which suggested a change in O-linked glycosylation (Fig. 2D–S9).

**Fig. 2:**
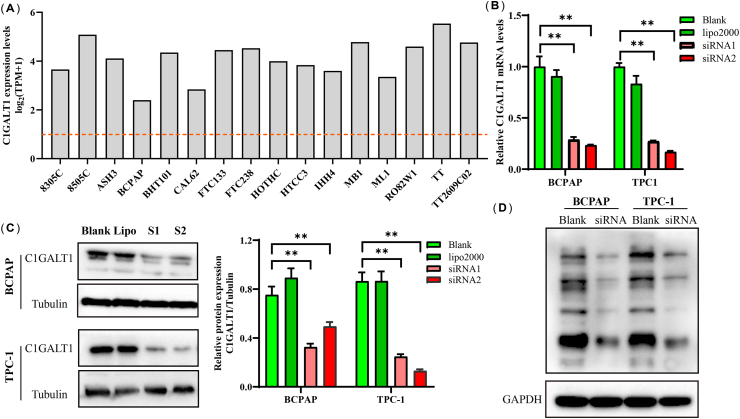
C1GALT1 expression in TC cells. **(A)** The C1GALT1 levels of 16 TC cell lines in the CCLE database. **(B, C)** The mRNA **(B)** and protein **(C)** expression of C1GALT1 in the siRNA group compared to the blank group. **(D)** The PNA binding lectin blotting. Data are shown as the mean ± SD (n = 5, **p < 0.01).

### 3.3 C1GALT1 promotes carcinogenesis in TC cells

The CCK-8, wound healing and Transwell assays were used to detect cell proliferation, migration and invasion in TC cells. As shown in Fig. 3A-C, we found that knockdown of C1GALT1 inhibited the carcinogenesis of TC at the proliferation, migration and invasion levels compared with the blank group. According to relevant studies, itraconazole (ITZ) is a specific inhibitor of C1GALT1 [15]. We discovered that the C1GALT1 protein levels were significantly reduced in the ITZ group in a dose-dependent manner (Fig. 3D–S10). Moreover, the migration and invasion abilities of TC cells were also remarkably suppressed by ITZ pretreatment (Fig. 3E and F). These findings suggested that C1GALT1 may serve as an oncogene in tumor growth and metastasis through cell migration and invasion.

**Fig. 3:**
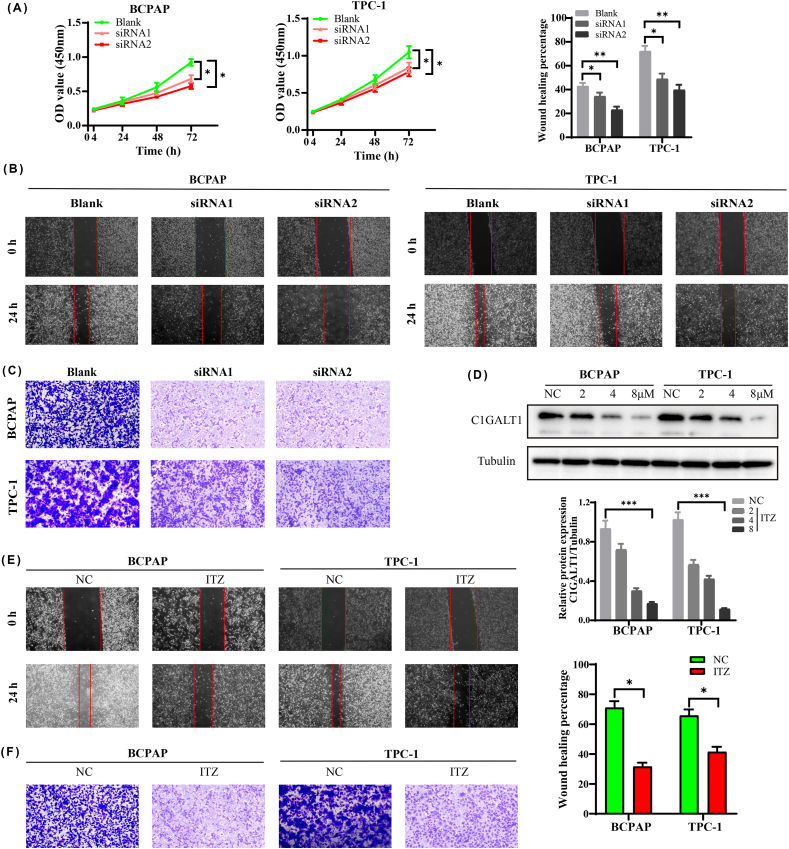
C1GALT1 promotes carcinogenesis in TC cells. **(A)** Cell proliferation as determined by CCK-8. **(B)** Cell migration as determined by wound healing assay. **(C)** Cell invasion as determined by Transwell assay. **(D)** The protein expression of C1GALT1 after treatment with ITZ for 48 h. **(E, F)** The inhibitory effect of ITZ (8 μM) on cell migration **(E)** and invasion **(F)** levels. Data are expressed as the mean ± SD (n = 5, *p < 0.05, **p < 0.01, ***p < 0.001).

### 3.4 MiR-141-3p suppresses the function of C1GALT1

To investigate the upstream regulatory mechanism of C1GALT1, we focused on miRNAs, the major gene regulators. Then, we predicted 4 key miRNA regulators through three different publicly available miRNA databases (TargetScan, TarBase and miRDB) (Fig. 4A, Table S4). In addition, some studies showed that has-miR-141-3p is closely associated with the progression of cancer [18]. Hence, we further explored the effect of miR-141-3p in targeting C1GALT1 of TC cells. The putative binding site between miR-141-3p and the C1GALT1 3′UTR is shown in Fig. 4B via the bioinformatics prediction of the TargetScan website. The dual-luciferase reporter assay also showed that the miR-141-3p mimics repressed the luciferase activity of wild-type C1GALT1 compared to mutant C1GALT1 (Fig. 4C). Furthermore, C1GALT1 expression levels were markedly decreased after transfecting miR-141-3p mimics into TC cell lines (Fig. 4D and E, S11).

**Fig. 4:**
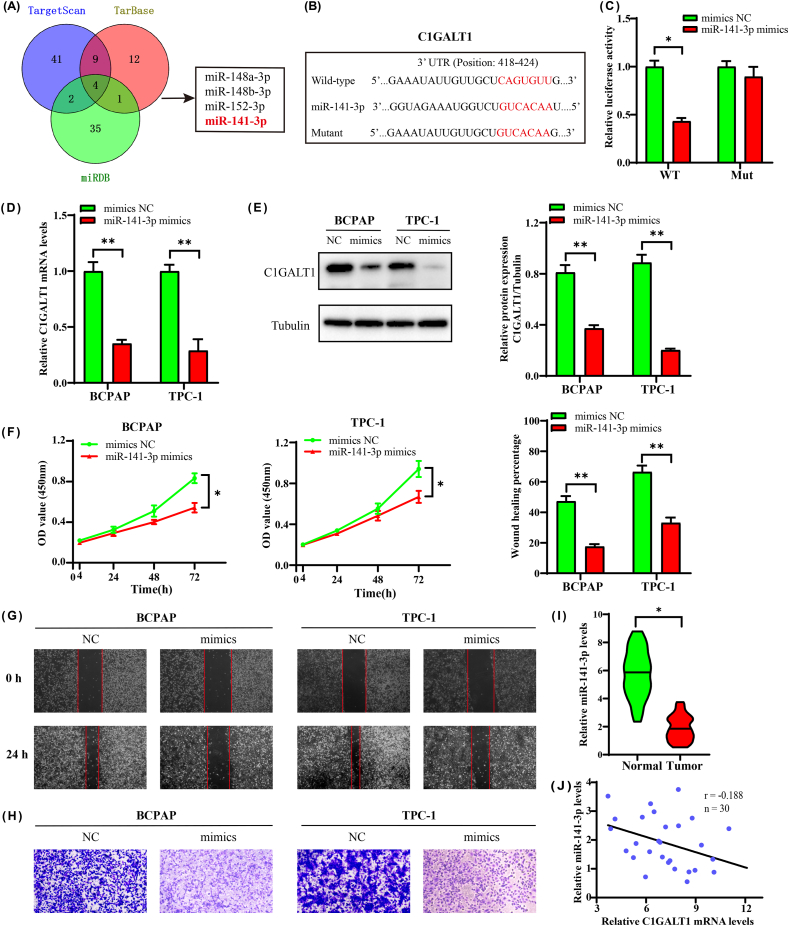
MiR-141-3p suppresses the function of C1GALT1. **(A)** Venn diagram of three publicly predicted miRNA databases. **(B)** The putative 3′UTR binding site between miR-141-3p and C1GALT1. **(C)** Dual-luciferase reporter assay. **(D, E)** The relative expression of C1GALT1 at the mRNA and protein levels. **(F–H)** Cell proliferation, migration and invasion assays. **(I)** Violin plot of relative miR-141-3p levels in TC tissues (n = 30). **(J)** The negative correlation of mRNA expression levels between miR-141-3p and C1GALT1 in clinical samples (n = 30). Data are presented as the mean ± SD (n = 5, *p < 0.05, **p < 0.01).

We next determined the cell proliferative ability. The growth rate of TPC-1 and BCPAP cells was obviously decreased in the miR-141-3p mimic group (Fig. 4F). Meanwhile, the results of wound healing and Transwell assays also showed consistently decreasing in the miR-141-3p mimic transfection group (Fig. 4G and H). To further confirm the correlation between miR-141-3p and C1GALT1, we examined the miR-141-3p levels in 30 clinical specimens by qPCR. As shown in Fig. 4I, the relative miR-141-3p expression levels were visibly reduced in tumor tissues. The mRNA levels of miR-141-3p and C1GALT1 were negatively correlated (Fig. 4J). Based on the abovementioned data, these results indicated that has-miR-141-3p was the upstream negative regulatory factor of C1GALT1 in TC.

### 3.5 C1GALT1 positively regulates GLUT1 expression

Although C1GALT1 is a specific enzyme that regulates glycans during the O-linked glycosylation reaction, the downstream regulatory element of C1GALT1 is still not reported in TC cells. Hence, we detected 68 energy metabolites, including carbohydrate metabolites, amino acids, nucleotide metabolites, organic acids, phosphate sugars and phosphoric acids, with BCPAP and its siRNA cell lines using UPLC-MS/MS. After data preprocessing and quality control analysis, 57 metabolites were identified, including 52 up-regulated and 5 down-regulated metabolites. The detailed data and heatmap of 57 metabolites are shown in Table S5 and Fig. S2. The top 10 up- or down regulated metabolites were also labeled in the dynamic distribution of metabolite levels (Fig. S3). Compared with the NC group, 7 significantly upregulated differential metabolites (DMs) were filtered with VIP >1 and FC ≥ 1.5 or ≤ 0.66 in the siRNA group (Fig. 5A, Table S6). Fig. 5B involves three typical DMs, which are closely related to glycosylation, such as 3-phosphoglycerate (glycolysis), isocitric aicd (tricarboxylic acid cycle) and glutamine (amino acid). The KEGG pathway enrichment of DMs contained transporters, purine metabolism, nucleotide metabolism and glyoxylate and dicarboxylate metabolism (Fig. 5C). The classification and differential abundance score of KEGG pathways are shown in Figs. S4 and S5, respectively.

**Fig. 5:**
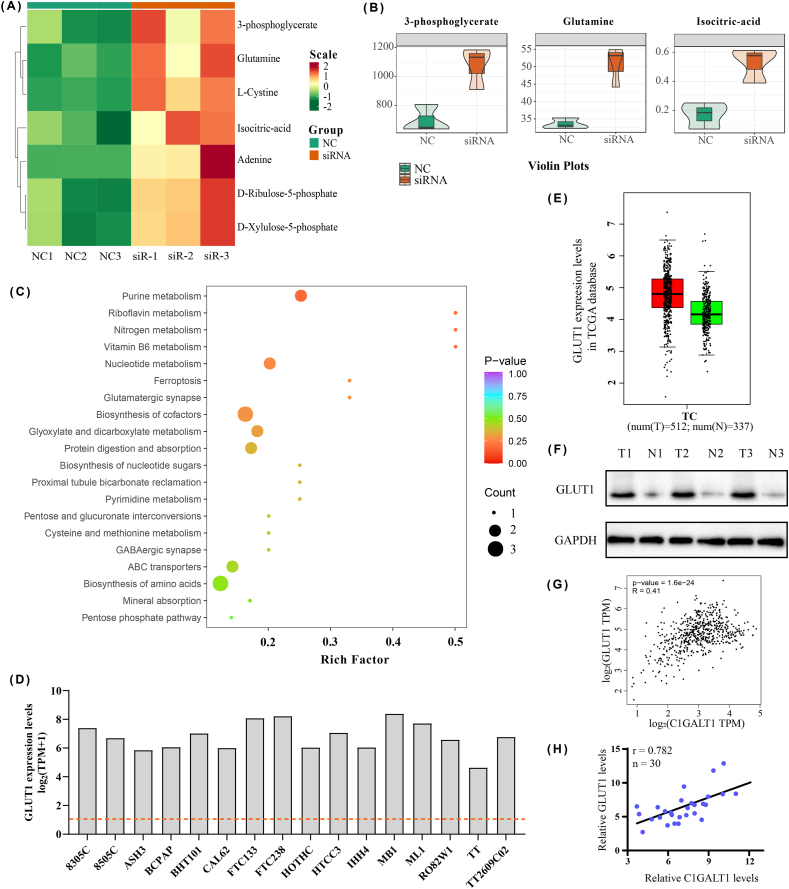
C1GALT1 positively regulates GLUT1 expression. **(A)** Heatmap of DMs in metabolomics (n = 3). **(B)** Violin plot of three typical DMs. **(C)** The KEGG enrichment pathway of DMs. **(D)** The higher expression of GLUT1 in TC cell lines according to the CCLE database. **(E)** The upper expression of GLUT1 in TC tissues by TCGA database. **(F)** GLUT1 protein levels in clinical specimens (n = 6). **(G, H)** The positive correlation of mRNA levels between GLUT1 and C1GALT1 in the Dependency Map database **(G)** and tissue samples (**H**, n = 30), respectively.

Owing to these findings, we turned our attention to glucose transporter 1 (GLUT1, SLC2A1), the key transporter of glucose and energy metabolism [12]. We found that GLUT1 mRNA levels had a sensible high expression in different TC cell lines through the CCLE database (Fig. 5D). The upper expression of GLUT1 was simultaneously exhibited in the tumor group by the Cancer Genome Atlas (TCGA) database (Fig. 5E). Additionally, we further ascertained that the protein levels of GLUT1 existed significantly increasing in clinical tumor tissues (Fig. 5F–S12). To understand the regulatory relevance between GLUT1 and C1GALT1 in TC, the mRNA levels were analyzed using the Dependency Map portal database and clinical specimens. As shown in Fig. 5G and H, the GLUT1(SLC2A1) had a markedly positive correlation with C1GALT1 in TC.

### 3.6 C1GALT1 increased the migration and invasion by GLUT1

To validate the regulatory role of GLUT1, we transfected BCPAP and TPC-1 cell lines with GLUT1 plasmid and C1GALT1 siRNA. The GLUT1 protein levels were inhibited in the siRNA group (Fig. 6A–S13). However, the suppression of GLUT1 expression was attenuated with the GLUT1 plasmid (*p* < 0.01, Fig. 6A). We further speculated that C1GALT1 affects growth and invasion activities by adjusting GLUT1. The results of proliferation and migration were augmented in cotransfected group compared with the siRNA group (Fig. 6B and C). Cell invasion also showed an obvious recovery in the GLUT1 group by Transwell assay (Fig. 6D). In short, these data indicated that GLUT1 was a downstream element of C1GALT1 in TC cells.

**Fig. 6:**
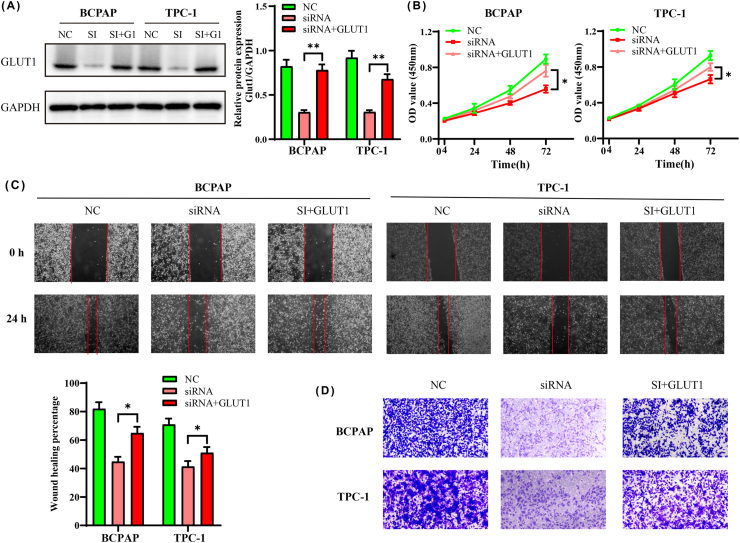
C1GALT1 increased migration and invasion by GLUT1. (**A**) The protein expression levels of GLUT1 after cotransfection with C1GALT1 siRNA and GLUT1 plasmid for 48 h. **(B**–**D)** Cell proliferation, migration and invasion assays. Data are presented as the mean ± SD (n = 5, *p < 0.05, **p < 0.01, SI: siRNA, G1: GLUT1).

## 4 Discussion

Relevant researches clarified that aberrant glycosylation is inherently associated with tumorigenesis and tumor progression. The activation of glycosylation reaction between oligo-mannose and hybrid N-glycans was associated with tumor metastasis [7]. Rømer reported that the glycosylation was induced by changing the levels of ZIP9 and Zn^2+^ [8].C1GALT1, a key regulated enzyme of O-glycosylation, plays an important role in the cancer development, metastasis and prognosis [19], such as breast cancer [20], bladder cancer [21], neuroblastoma [22^]^ and endometrial cancer [23^]^. However, the biological function and potential molecular mechanism of C1GALT1 in thyroid cancer have not yet been reported. This study found that C1GALT1 was higher expression in TC clinical specimens and cell lines. We further confirmed that C1GALT1 was negatively regulated by miR-141-3p and induced cell growth and metastasis by adjusting GLUT1 expression levels. Similarly, a recent study elucidated that suppression of C1GALT1 caused the changing of protein glycosylation and the reducing cell adhesion and migration in colon cancer cells [22]. Lin explained that the loss of C1GALT1 inhibited tumor growth in the mouse models of head and neck cancer [23]. Other studies also demonstrated that C1GALT1 was used to as a biomarker to identify different cancers [[21], [24], [25]]. C1GALT1 levels were consistent with FGFR3 expression levels, a specific bladder protein, in bladder cancer [21]. Depletion of C1GALT1 also induced the expression of invasive proteins in endometrial cancer, increasing the invasion ability of cancer cells [25]. To date, this is the first study to declare the positive regulatory relationship between C1GALT1 and GLUT1. These results provide data support for the molecular regulation of glucose metabolism in TC, and warn the excessive intake of carbohydrate in cancer patients. However, the metabolic mode and converted mechanism of glucose in TC cells need to be further explored, including verification in animal experiments. Therefore, C1GALT1 may be as a probable biomarker in TC progression.

It has been reported that miRNAs play a crucial role in cell biology, including cell proliferation, differentiation and motility [26,27]. The gain or loss of miRNAs would largely disturb the balance of cell growth, adhesion and metastasis, especially in malignant tumors [[28], [29], [30]]. Therefore, we ascertained the negative correlation between miR-141-3p and C1GALT1 through public miRNA databases and luciferase reporter assay. The results of proliferation, migration and invasion also indicated that miR-141-3p attenuated cell abilities by decreasing C1GALT1 levels in TC cell lines. Our findings were consistent with the function of miR-141-3p in other cancers [31,32]. For example, Mo exposed that the exosomal miR-141-3p mediated tumor-stroma interactions and cancer metastasis by activating the YAP1/GROα/CXCR signaling cascade in ovarian cancer [31]. Deregulated of miR-141-3p expression was also related to cancer recurrence in breast cancer patients [24]. Stable miR-141-3p overexpression could alter the DNA damage responses, signaling pathways and cell cycle, even as a biomarker in T-prolymphocytic leukemia [32].

Recently, many studies suggested that glucose serve as an important compound in tumor growth, including cell metabolic reprogramming, tumor microenvironment and the development of anticancer agents [33–34]. Warburg effect provides some tangible advantage to cancer cells, such as a higher rate of ATP production, plenty intermediates required for cell biosynthesis and a low immunity microenvironment [35]. The key targeted proteins and intermediates in glucose metabolism can be used to develop glycolytic inhibitors as anticancer drugs [34]. GLUT1 is widely known as a key transporter in regulating the physiological and pathological processes of tumors in the glucose metabolic pathway [36,37]. One study illustrated that the knockout of DHHC9 inhibited GLUT1 levels in glioblastoma, and further impaired glycolysis, cell proliferation and tumorigenesis [38]. Chen showed that METTL3 induced GLUT1 translation to promote glucose uptake and colorectal cancer development [39]. The GLUT1 expression level of tumor-associated neutrophils disrupted cell growth and radiotherapy resistance in lung cancer [40]. Although C1GALT1 is closely related to glycosylation and glucose metabolism, its effect on GLUT1 has not been thoroughly studied in various cancers. In this study, we revealed that GLUT1 expression was repressed in the C1GALT1 siRNA group and partially increased cellular proliferation, migration and invasion abilities via the GLUT1 plasmid. Thus, GLUT1 was a downstream target of C1GALT1 in TC.

The regulated correlation between C1GALT1 and GLUT1 has been presented, whereas in fact there are several limitations in the current work aspects that need further study. For instance, the detailed mechanisms of downstream glucose-related metabolites affecting cell proliferation and invasion need further investigation. The variation in the C1GALT1-mediated tumor microenvironment and signaling pathways requires further exploration. Overall, our study clarified that C1GALT1 was overexpressed in thyroid cancer cell lines and tissues. In addition, the growth and metastasis induced by C1GALT1 could be mitigated by miR-141-3p and GLUT1 in TC cells.

## 5 Conclusion

All in all, we found lower levels of miR-141-3p, higher levels of C1GATL1 and GLUT1, and the interactions between them in TC. Meanwhile, we highlight the importance of the miR-141-3p/C1GALT1/GLUT1 regulatory axis during thyroid cancer progression. The C1GALT1 could as a novel prognostic biomarker or design as a drug target for TC treatment. The experimental data of this study may be used for the development of innovative therapeutic regimens for thyroid cancer.

## Ethics statement

This study was approved by the Ethics Committee of Henan Provincial People's Hospital, with the approval number: 2021-84. All patients provided informed consent to participate in the study. Clinical specimens were collected from Henan Provincial People's Hospital.

## Funding

This study was supported by the 10.13039/501100001809National Natural Science Foundation of China [grant number: 82100923], the key scientific and technological projects of Henan Province [grant number: 232102310159], the scientific and technological projects of medicine in Henan Province [grant number: LHGJ20230043] and the international medical exchange foundation of China [grant number: Z-2021-46-2101-2023].

## Data availability statement

Data included in article/supp. material in article.

## CRediT authorship contribution statement

**Li Huang:** Writing – original draft, Methodology. **Zhen Li:** Data curation. **Ziguang Xu:** Formal analysis. **Ruili Yu:** Funding acquisition, Data curation. **Chao Ding:** Investigation. **Tingyi Sun:** Software. **Lingfei Kong:** Validation. **Zhengchao Xia:** Writing – review & editing, Funding acquisition.

## Declaration of competing interest

The authors declare that they have no known competing financial interests or personal relationships that could have appeared to influence the work reported in this paper.

## Figures and Tables

**figs1:**
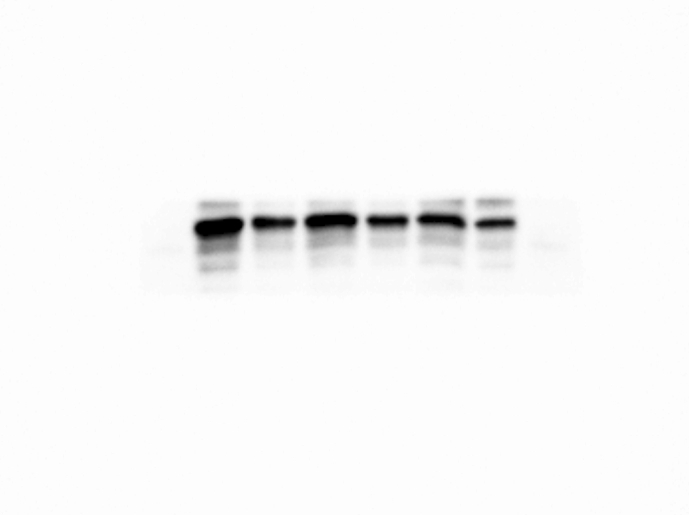


**figs2:**
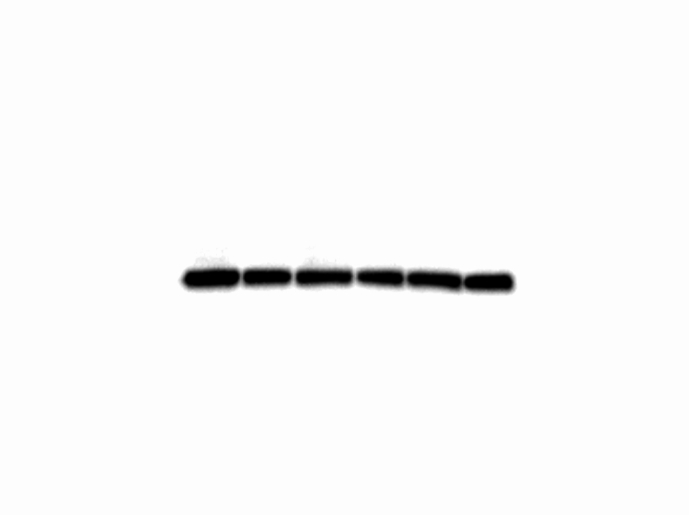


**figs3:**
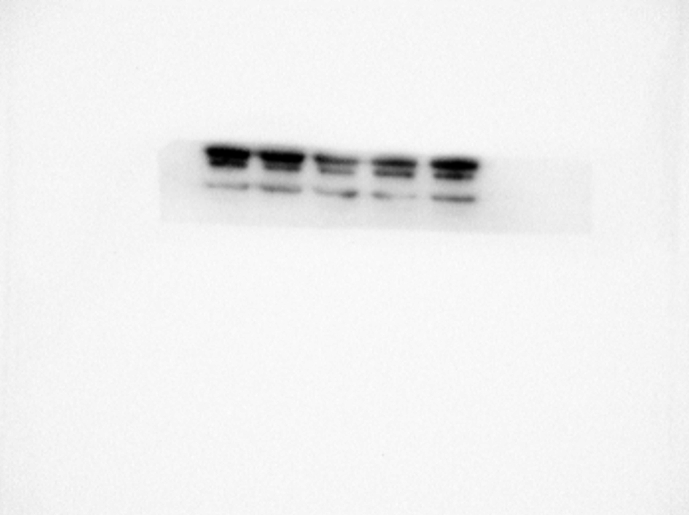


**figs4:**
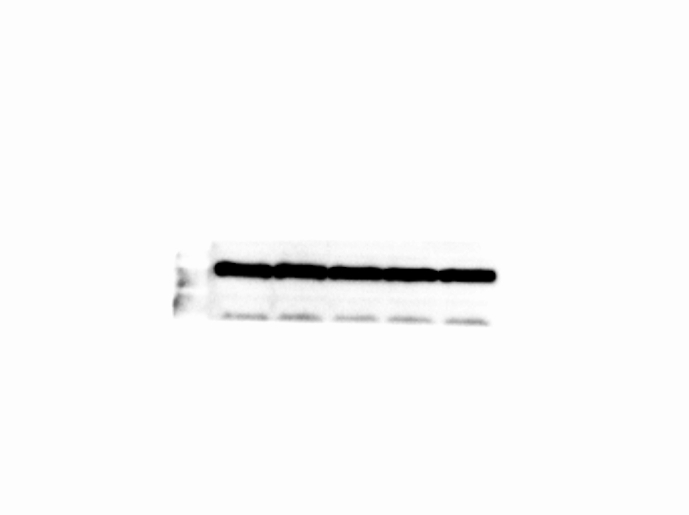


**figs5:**
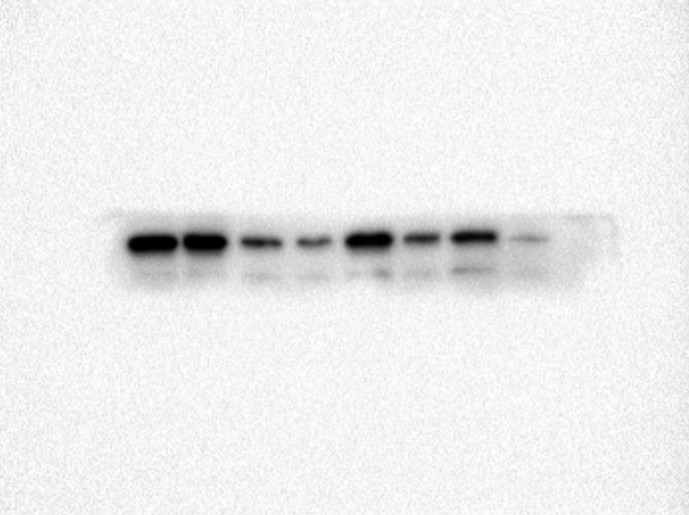


**figs6:**
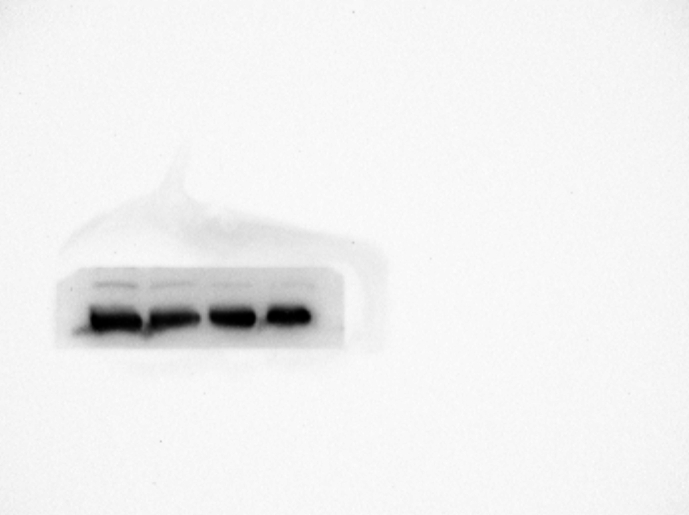


**figs7:**
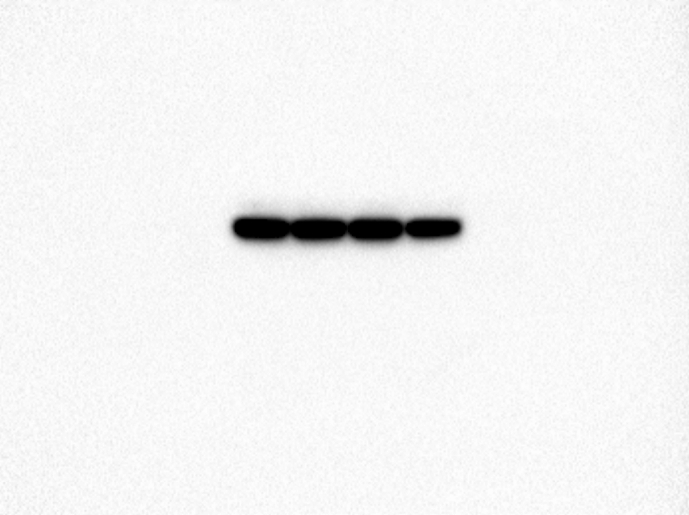


**figs8:**
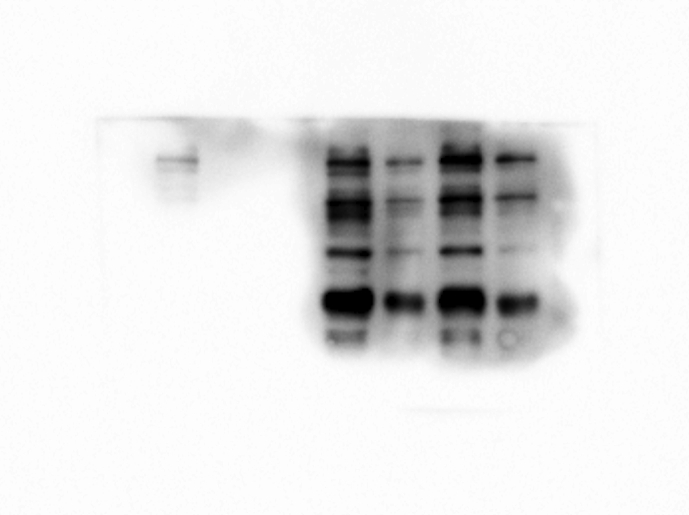


**figs9:**
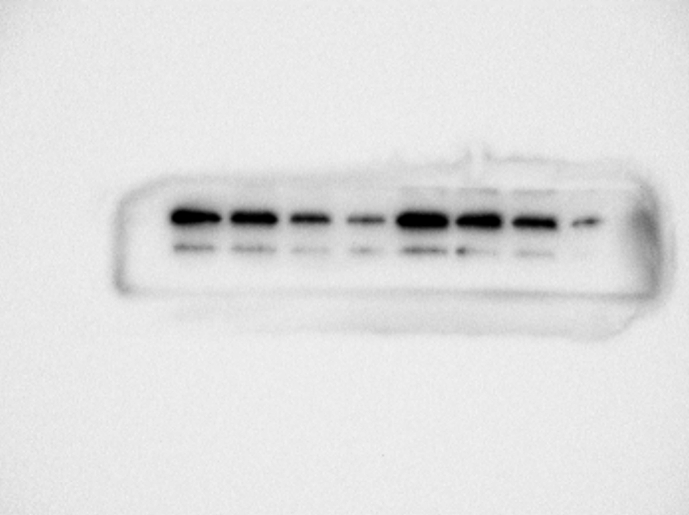


**figs10:**
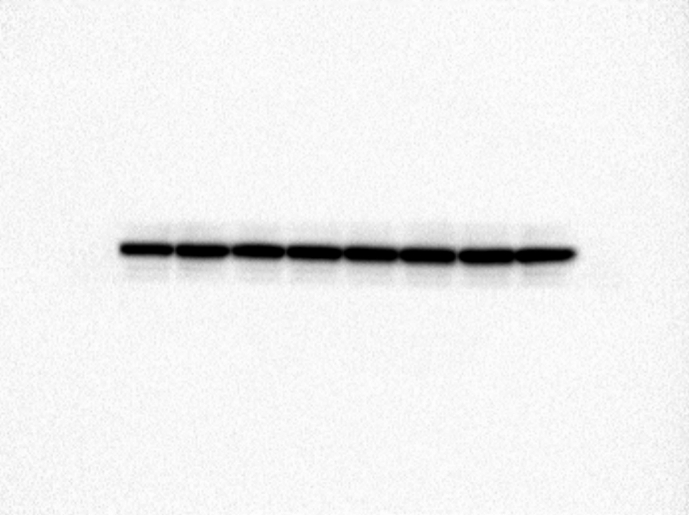


**figs11:**
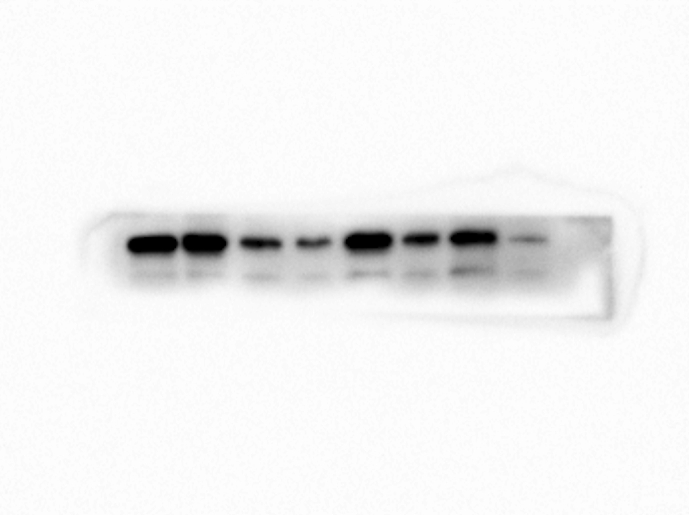


**figs12:**
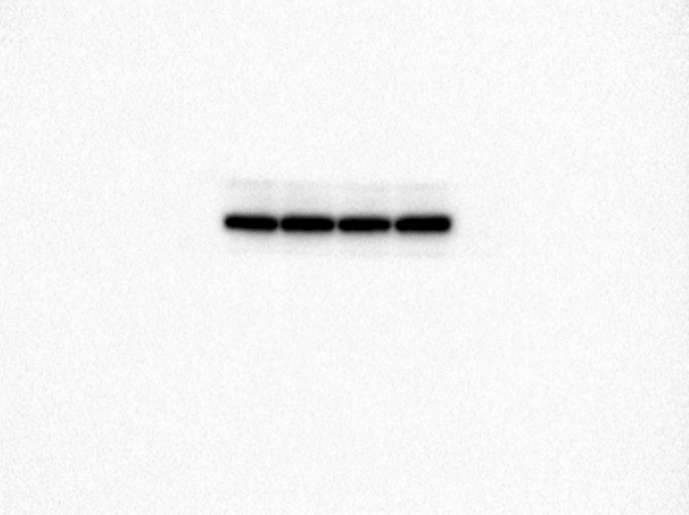


**figs13:**
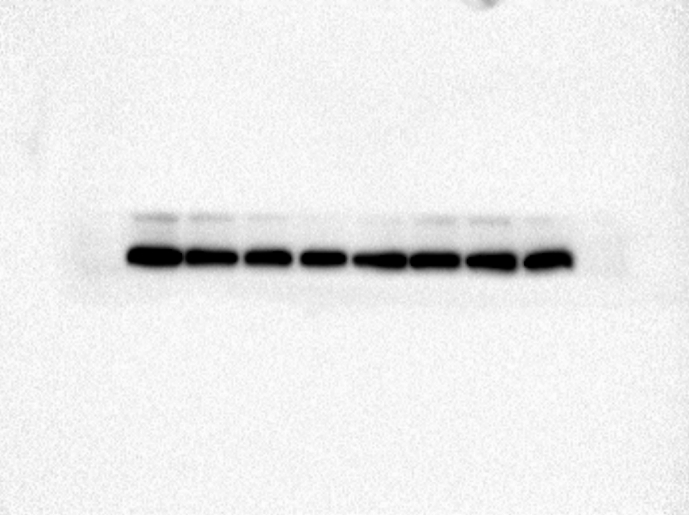


**figs14:**
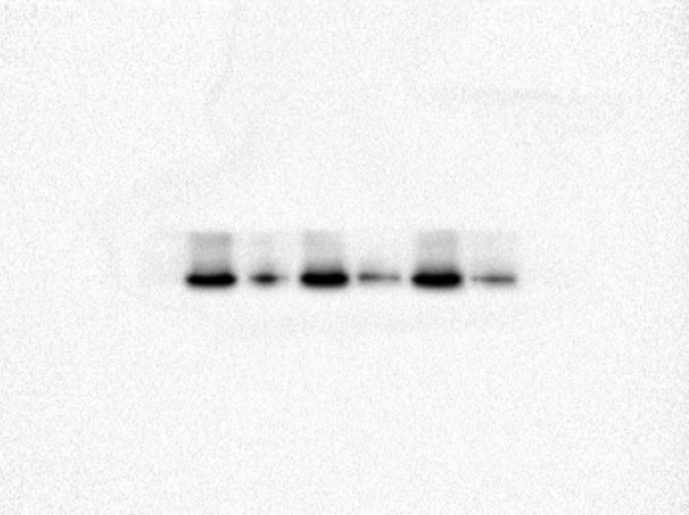


**figs15:**
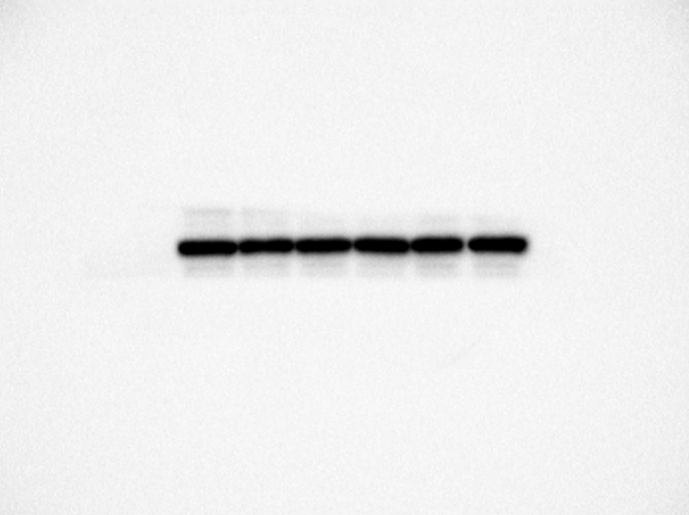


**figs16:**