# Supplementary material for: C1GALT1 induces the carcinogenesis of thyroid cancer through regulation by miR-141-3p and GLUT1
Source: Heliyon. 2024 May 24;10(11):e31778. doi: 10.1016/j.heliyon.2024.e31778 (PMC11153184; doi:10.1016/j.heliyon.2024.e31778)
Supplement: Multimedia component 1 [file mmc1.docx]

***Supplementary Materials***

**C1GALT1 induces the carcinogenesis of thyroid cancer through regulation by miR-141-3p and GLUT1**

Li Huang^1^, Zhen Li^1^, Ziguang Xu^1^, Ruili Yu^1^, Chao Ding^2^, Tingyi Sun^1^, Lingfei Kong^1^, Zhengchao Xia^3*^

*** Correspondence:**

Zhengchao Xia, zcxia@zzu.edu.cn

**Quantitative analysis of western blotting**

Firstly, the image format is converted to 8-bit by Type in the Image. Secondly, the peak area of bands was shown by Select First Lane and Plot Lanes in Gels of Analyze. Thirdly, we split the individual peaks with a line tool and measure the area with the magic wand tool.

**Quantitative analysis of cell migration assay**

First, the image format is converted to 8-bit by Type in the Image. Then, the image increased contrast by Enhance Contrast (Saturated pixels: 6%, normalize), smoothing, and highlighting the cell edges by Find Edges in Process. Finally, the area of migration was displayed by regulating the threshold by Adjust in Image, and measured the area in Analyze after using the magic wand tool.

**Detection of targeted energy metabolites**

HPLC-grade acetonitrile (ACN) and methanol (MeOH) were purchased from Merck (Darmstadt, Germany). MilliQ water (Millipore, USA) was used in all experiments. All of the standards were purchased from Sigma-Aldrich (St. Louis, USA). Formic acid was bought from Sigma-Aldrich (St. Louis, USA). The stock solutions of standards were prepared at the concentration of 1 mg/mL in MeOH and other solutions. All stock solutions were stored at -20°C. The stock solutions were diluted with MeOH to working solutions before analysis.

The sample was thawed on ice, 100 μL of ultrapure water extract was added to resuspend the cell pellet. Divide 50 μL cell suspension and add 200 μL of methanol (precooled at -20°C) and vortexed for 2 min under the condition of 2500 r/min. The sample was frozen in liquid nitrogen for 5 min, removed on ice for 5 min, and vortexed for 2 min. The previous step was repeated for 3 times. The sample was centrifuged at 12000 r/min for 10 min at 4°C. Take 200 μL of supernatant into a new centrifuge tube and place the supernatant in -20°C refrigerator for 30 min. The supernatant was centrifuged at 12000 r/min for 10 min at 4°C. After centrifugation, transfer 180 μL of supernatant through Protein Precipitation Plate for further LC-MS analysis. The left 50 μL cell suspension was frozen and thawed for 3 times, centrifuged at 12,000 r/min for 10 min, and the supernatant was taken to determine the protein concentration by BCA Protein Assay kit.

The sample extracts were analyzed using an UPLC-ESI-MS/MS system (Waters ACQUITY H-Class and QTRAP® 6500). The analytical conditions were as follows: HPLC column(ACQUITY UPLC BEH Amide, 2.1×100 mm, 1.7 μm); solvent system (A: water with 10mM Ammonium acetate and 0.3% Ammonium hydroxide; B: 90% acetonitrile/water(V/V)); the gradient methods (95% B (0-1.2 min), decreased to 70% B (8 min), 50% B (9-11 min), ramped back to 95% B (11.1-15 min)); flow rate (0.4 mL/min); temperature at 40°C; injection volume at 2 μL. The LC-MS/MS System equipped with an ESI Turbo Ion-Spray interface and controlled by Analyst 1.6 software. The ESI source operation parameters were as follows: ion source, turbo spray; source temperature at 550°C; ion spray voltage (IS) 5500 V (Positive), -4500 V (Negative); curtain gas (CUR) were set at 35.0 psi; DP and CE for individual MRM transitions was done with further DP and CE optimization. A specific set of MRM transitions were monitored for each period according to the plant hormones eluted within this period.

Unsupervised PCA (principal component analysis) was performed by statistics function within R. The data was unit variance scaled before unsupervised PCA. The HCA (hierarchical cluster analysis) results of samples and metabolites were presented as heatmaps with dendrograms, while Pearson correlation coefficients (PCC) between samples were calculated by R package.

Significantly regulated metabolites between groups were determined by VIP and absolute Log_2_FC. VIP values were extracted from OPLS-DA result, which also contain score plots and permutation plots, was generated using R package MetaboAnalystR. The data was log transform (log_2_) and mean centering before OPLS-DA. In order to avoid overfitting, a permutation test (200 permutations) was performed. Identified metabolites were annotated using KEGG compound database, annotated metabolites were then mapped to KEGG Pathway database. Pathways with significantly regulated metabolites mapped to were then fed into MSEA (metabolite sets enrichment analysis), the significance was determined by hypergeometric test’s *P*-Values.

**Supplementary Tables**

**Table S1**. The sequences of all the primers.

| **Name** | **Oligonucleotide sequence (5' - 3')** |
| --- | --- |
| C1GALT1 | F: CAAAATACGACCCTGAAGAAC |
|  | R: GCATCTCCCCAGTGCTAAGTC |
| GLUT1 | F: CAGGCTTCGTGCCCATGTAT |
|  | R: ACAGATCCGAGAGCCACTGA |
| miR-141-3p | F: GCCGAGTAACACTGTCTGGT |
|  | R: CTCAACTGGTGTCGTGGAGT |
| U6 | F: CTCGCTTCGGCAGCACA |
|  | R: AACGCTTCACGAATTTGCGT |
| GAPDH | F: AGCCACATCGCTCAGACAC |
|  | R: GCCCAATACGACCAAATCC |

**Table S2**. The antibodies of all proteins.

| **Name** | **Description** | **Product code** | **Lot number** |
| --- | --- | --- | --- |
| C1GALT1 | rabbit polyclonal | A7590 | 1155110201 |
| GLUT1 | rabbit polyclonal | 21829 | 00115893 |
| PNA | biotinylated | B-1075 | ZH0422 |
| Streptavidin | peroxidase | SA-5014 | ZH0506 |
| Tubulin | rabbit polyclonal | 22034 | 00101141 |
| GAPDH | mouse monoclonal | 60004 | 10020246 |

**Table S3**. The raw data of three lectins (UEA I, VVL and PNA).

Table S3 includes column, row, name, ID, mean and *p*-value.

**Table S4**. Predicted miRNAs by **three different publicly miRNAs database**.

The analysis was processed by Venny 2.1 software, including 56 miRNAs in TargetScan, 26 miRNAs in TarBase, 42 miRNAs in miRDB database.

**Table S5**. The information of identified metabolites.

Table S5 contains 57 energy metabolites. It also includes compounds, class (Amino acids, carbohydrate metabolomics, coenzyme and vitamins, nucleotide, organic acid, Phosphoric acids), molecular weight, ion mode, ionization model, formula, the peak area of different samples, the ID of compound, HMDB and CAS, and kegg map.

**Table S6**. The related information of different metabolites.

Table S6 contains 7 up-regulated metabolites. It also includes compounds, class, the peak area, VIP, P-value, FDR, Fold change and type of level.

**Supplementary Figures**


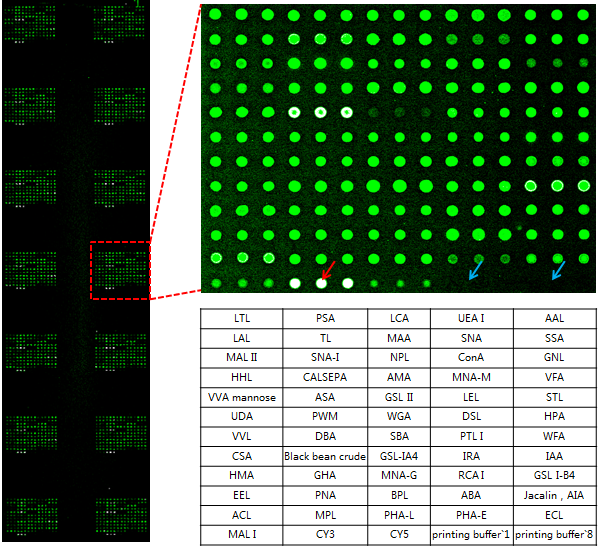


**Figure S1**. The quality control of lectin microarray analysis.

The left image is the global scan of the chip, the upper right image is the enlarged image of the local part of the chip (Block 8), and the lower right image is the corresponding lectins, among which there are 56 lectins, and each lectin is repeated by 3 dots. According to the QC results, the red arrow is the positive control point (Cy3), and the blue arrow indicates the negative control point (printing buffer). The preparation of this batch of chips is qualified.


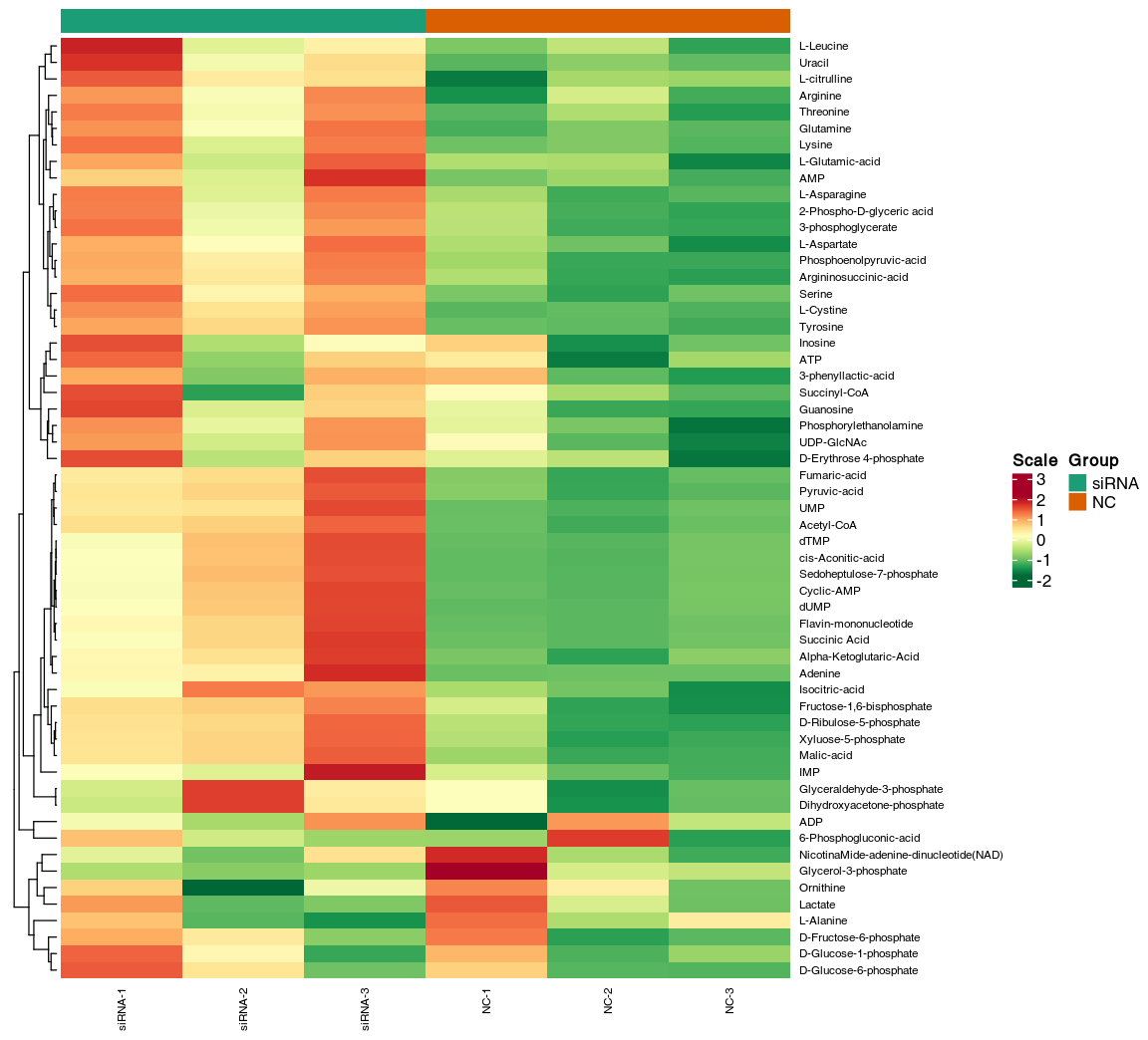


**Figure S2**. The cluster analysis heatmap of 57 energy metabolites.

The horizontal coordinate is the sample name, and the vertical coordinate is the metabolite information. Red and green indicated high and low expression, respectively.


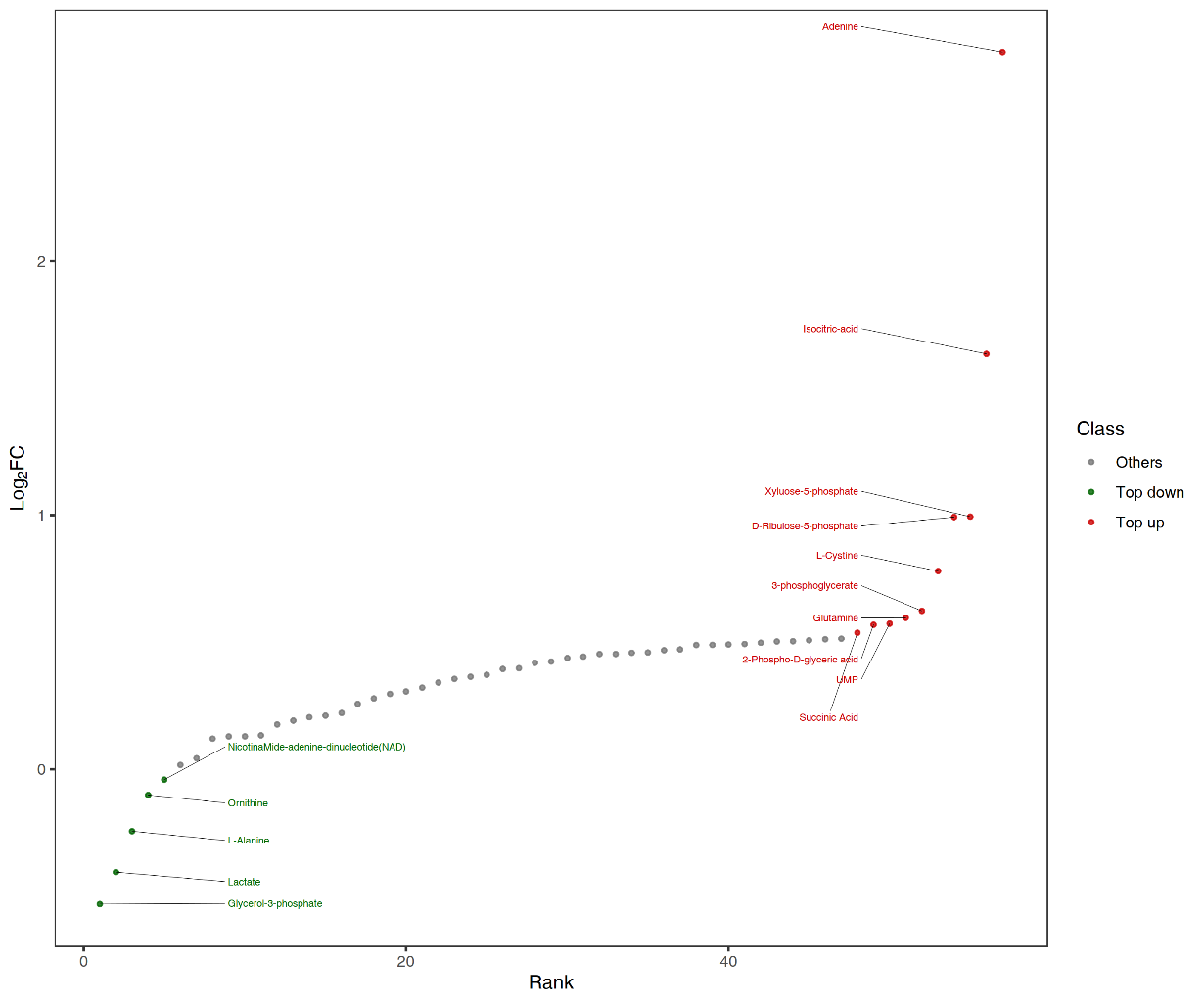


**Figure S3.** The dynamic distribution of metabolite levels.

The horizontal coordinate represents the cumulative amounts of metabolites, and the vertical coordinate represents log_2_ fold change. Each dot is a metabolite. And red and green indicated the top 10 up- or down-regulated, respectively.


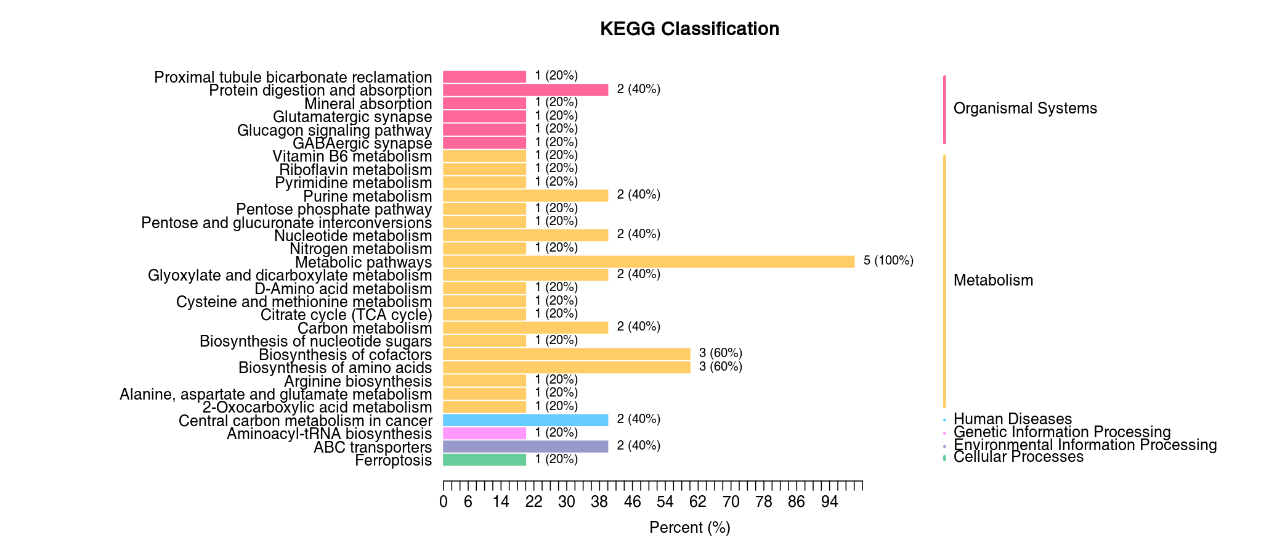


**Figure S4**. The KEGG classification plot of differential metabolites.

The figure displayed the KEGG annotations and pathway enrichment analysis of differential metabolites. The left side showed the name of each pathway. The numbers in the histogram indicated the number and proportion of differential metabolites enriched into this pathway.


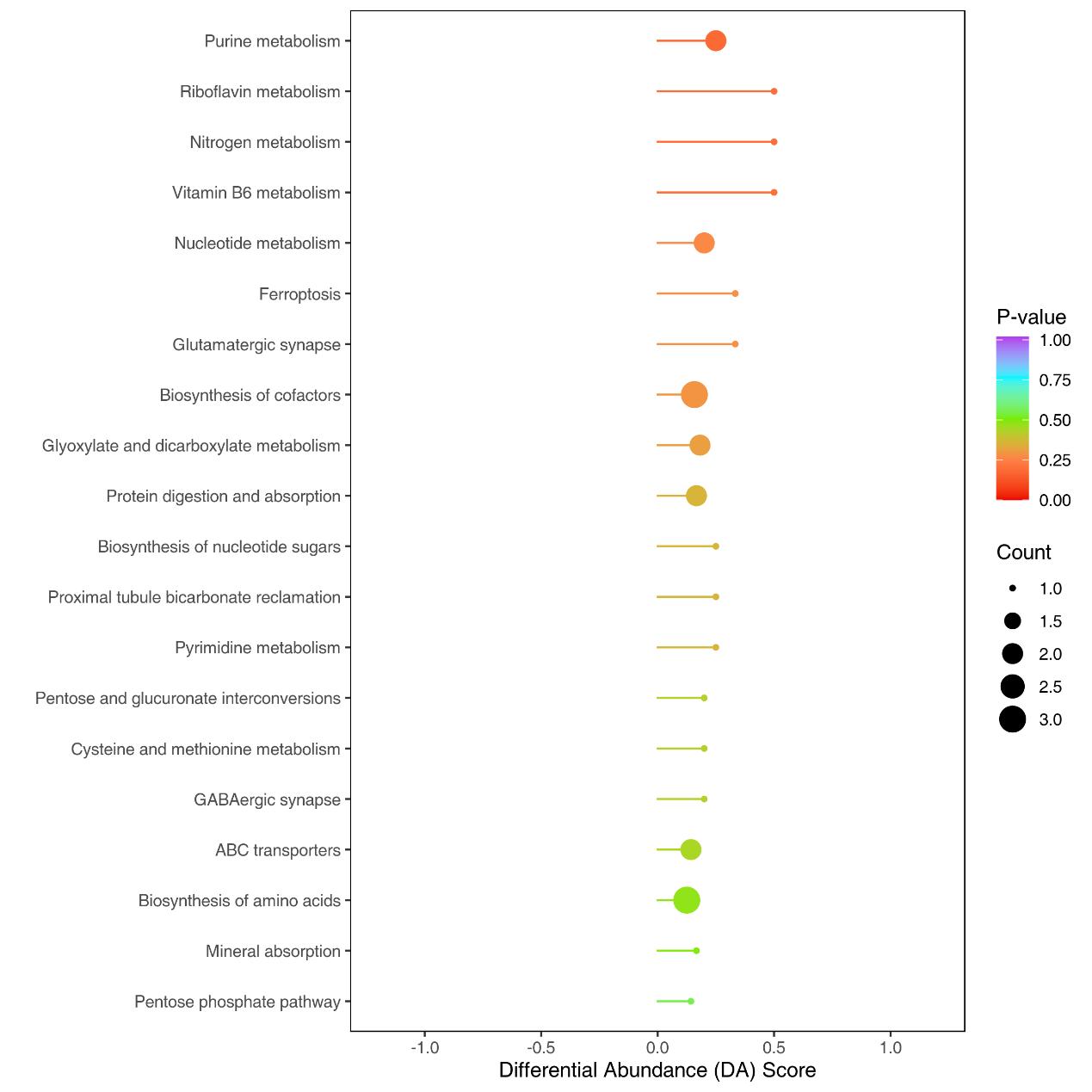


**Figure S5**. The differential abundance (DA) score plot of differential metabolites.

The ordinate represented the name of difference pathway, and the abscissa represented the DA score. The DA score reflected the overall change in all metabolites of the metabolic pathway. Score 1 and -1 indicated up- or down-regulated trends in differential metabolite expression in this pathway, respectively. The segment length indicated the absolute value of DA score. The dot size and color indicated the number of differential metabolites and the P-value of this pathway.


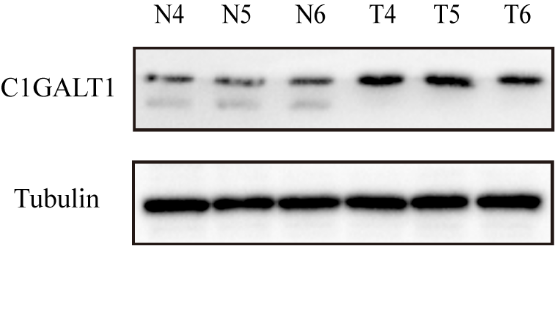


**Figure S6**. **C1GALT1 protein expression levels (n=3, N: normal, T: tumor).**

**Figure S7. The uncropped image of figure 1D.**

The first, third and fifth strips were cancer tissue samples. The second, fourth and sixth strips were corresponding adjacent benign tissues.

**Figure S8. The uncropped image of figure 2C.**

**For BCPAP:** the first to fourth strips were blank, lipo2000, siRNA1 and siRNA2 group, respectively. The fifth strip were siRNA3 group, but its inhibitory effect is not obvious.

**For TPC:** the first to fourth strips were blank, lipo2000, siRNA1 and siRNA2 group, respectively. The fifth to eighth strips of Fig.2C-TPC-C1GALT1 were Fig. 4E-C1GALT1.

**Figure S9. The uncropped image of figure 2D.**

The first to fourth strips were blank and siRNA group of BCPAP and TPC-1 cells, respectively.

**Figure S10. The uncropped image of figure 3D.**

The first to fourth strips were normal control, 2 μM, 4 μM and 8 μM ITZ group in BCPAP cells. The fifth to eighth strips were corresponding group in TPC-1 cell.

**Figure S11. The uncropped image of figure 4E.**

The fifth to eighth strips of Fig.4E-C1GALT1 were normal control and miRNA mimics group of BCPAP and TPC-1 cells, respectively. The first to fourth strips of Fig.4E-C1GALT1 were figure 2C-TPC-C1GALT1.

**Figure S12. The uncropped image of figure 5F.**

The first, third and fifth strips were cancer tissue samples. The second, fourth and sixth strips were corresponding adjacent benign tissues.

**Figure S13. The uncropped image of figure 6A.**

The first to third strips were normal control group, siRNA group and cotransfection with C1GALT1 siRNA and GLUT1 plasmid group in BCPAP cell. The fourth to sixth strips were corresponding group in TPC-1 cell.
